# Transcriptomic Profiles Reveal Downregulation of Low-Density Lipoprotein Particle Receptor Pathway Activity in Patients Surviving Severe COVID-19

**DOI:** 10.3390/cells10123495

**Published:** 2021-12-10

**Authors:** Ivan Vlasov, Alexandra Panteleeva, Tatiana Usenko, Mikhael Nikolaev, Artem Izumchenko, Elena Gavrilova, Irina Shlyk, Valentina Miroshnikova, Maria Shadrina, Yurii Polushin, Sofya Pchelina, Petr Slonimsky

**Affiliations:** 1 Institute of Molecular Genetics of National Research Center “Kurchatov Institute”, 123182 Moscow, Russia; invlasov@mail.ru (I.V.); shadrina@img.ras.ru (M.S.); 2 Pavlov First Saint-Petersburg State Medical University, 197022 Saint-Petersburg, Russia; aleksandra9122@mail.ru (A.P.); usenko_ts@pnpi.nrcki.ru (T.U.); almaflex@mail.ru (M.N.); egavrilova70@mail.ru (E.G.); irina_shlyk@mail.ru (I.S.); miroshnikova_vv@pnpi.nrcki.ru (V.M.); polushin1@gmail.com (Y.P.); sopchelina@hotmail.com (S.P.); 3 Petersburg Nuclear Physics Institute Named by B.P. Konstantinov of National Research Center “Kurchatov Institute”, 188300 Saint-Petersburg, Russia; artemiz98@yandex.ru; 4 Kurchatov Genome Center—PNPI, 188300 Saint-Petersburg, Russia

**Keywords:** peripheral blood mononuclear cells, COVID-19, transcriptome, GO ontology, LDL particle receptor activity, leukocyte differentiation, cargo receptor activity

## Abstract

To assess the biology of the lethal endpoint in patients with SARS-CoV-2 infection, we compared the transcriptional response to the virus in patients who survived or died during severe COVID-19. We applied gene expression profiling to generate transcriptional signatures for peripheral blood mononuclear cells (PBMCs) from patients with SARS-CoV-2 infection at the time when they were placed in the Intensive Care Unit of the Pavlov First State Medical University of St. Petersburg (Russia). Three different bioinformatics approaches to RNA-seq analysis identified a downregulation of three common pathways in survivors compared with nonsurvivors among patients with severe COVID-19, namely, low-density lipoprotein (LDL) particle receptor activity (GO:0005041), important for maintaining cholesterol homeostasis, leukocyte differentiation (GO:0002521), and cargo receptor activity (GO:0038024). Specifically, PBMCs from surviving patients were characterized by reduced expression of PPARG, CD36, STAB1, ITGAV, and ANXA2. Taken together, our findings suggest that LDL particle receptor pathway activity in patients with COVID-19 infection is associated with poor disease prognosis.

## 1. Introduction

Coronavirus disease (COVID-19) is caused by infection with the severe acute respiratory syndrome coronavirus 2 (SARS-CoV-2) from the HCoV family of coronaviruses [1]. The first cases of this disease were described in China in the province of Wuhan, but by the end of January 2020, COVID-19 had been diagnosed in almost 12,000 people from 27 countries, and 259 patients had died. On 11 March 2020, the World Health Organization announced the COVID-19 pandemic—by this time the disease had been detected in 126 countries worldwide in more than 125,000 people [2]. For current information on COVID-19 incidence, visit the Center for Systems Science and Engineering (CSSE) at Johns Hopkins University (JHU), https://coronavirus.jhu.edu/map.html (accessed on 3 December 2021) Such a rapid development of the infectious process is associated with the airborne spread of SARS-CoV-2 and a short incubation period (in most cases, from 3 to 7 days clinical signs of the disease are observed, primarily fever and impaired sense of smell) [3].

Angiotensin-converting enzyme 2, known as the ACE2 receptor protein, serves as a target or entry point for SARS-CoV-2 [4,5,6]. The binding of the virus spike protein S1 to this receptor leads to rapid internalization of the virus by ACE2-expressing cells, primarily epithelial cells of the upper respiratory tract, alveolocytes, and enterocytes of the small intestine [7]. Individual differences in the level of expression of ACE2 can influence the risk of developing COVID-19 and the severity of the disease [8]. As ACE2 plays the role of a negative regulator of the activity of the entire renin–angiotensin–aldosterone system (RAAS), the partial dysfunction of ACE2 caused by the interaction with the viral protein S1 can lead to damage to various organs and tissues; from the lungs as the primary target of damage to the nervous tissue [9].

New SARS-CoV-2 viral particles maturing in infected epithelial cells of the alveoli through exocytosis enter the lung tissue, which results in the release of many proinflammatory cytokines (cytokine storm), infiltration of the lung tissue by macrophages, neutrophils, and T-cells, and the development of acute respiratory distress syndrome (ARDS). ARDS is characterized by severe impairment of lung function with decreased blood oxygen saturation [3,10]. The severity of the cytokine storm determines the clinical severity of the disease, and a pronounced increase in the level of proinflammatory cytokines is associated with the development of multiorgan damage to various organs and systems.

The further course of the pathological process in critically ill patients depends on several factors, such as age, the presence of comorbid conditions, and the level of D-dimers and lactate in peripheral blood [11]. The possibility that the genetic background can also influence the outcome of COVID-19 is also proposed. Inherited susceptibility can explain an individual response to viral infection in some human viral diseases [12]. One of the best examples is herpes simplex virus encephalitis, which in up to 7% of cases is caused by mutations in the genes for TLR3 or DBR1 [13]. COVID-19 is a completely new disease. Many studies analyzing a genetic basis of predisposition to COVID-19 on exome and genome levels are now being conducted [12]. However, definite genetic variants explaining severe COVID-19 cases have not yet been elucidated.

One of the possible approaches to search for such factors is analysis of the transcriptome and proteome of the peripheral blood of patients because each pathogen creates a unique transcriptional landscape based on the individual genome. A few studies have been published over the past year exploring proteomic and whole-transcriptomic RNA sequencing, including miRNAs and lncRNAs in various groups of patients with COVID-19 [14,15,16]. However, in all studies mentioned above the comparison was conducted between infected patients and controls, without assessing the disease outcome.

For a more accurate understanding of the course of the pathological process in patients with severe COVID-19, it is necessary to continue the search for factors that affect the course of the pathological process and the possibility of a favorable outcome in critically ill patients. To identify the genetic factors for potential recovery from severe COVID-19 requiring intensive therapy and resuscitation, we have, for the first time to our knowledge, conducted a study of RNA sequencing in patients at the time of admission to the intensive care unit with a prospective assessment of the outcome of the disease in an acute period (30 days). This study allowed the identification of genes differentially expressed in peripheral blood mononuclear cells (PBMCs) of patients with severe COVID-19 with different outcomes.

## 2. Materials and Methods

### 2.1. Patients

From 1 November 2020 to 25 February 2021, 200 patients were admitted to the Intensive Care Unit (ICU) of the Pavlov First State Medical University of St. Petersburg with a diagnosis of COVID-19 according to NEWS\NEWS2 criterium [17,18], of whom 44 met inclusion criteria. The inclusion criteria were Russian ethnicity, age between 40 and 80 years with the absence of chronic comorbidities such as cancer, cerebrovascular diseases, heart failure, or renal failure. All patients were SARS-CoV-2 positive and had severe pneumonia. SARS-CoV-2 infection was confirmed by reverse-transcriptase polymerase chain reaction. Patients were followed up for 30 days: 14 patients died and 30 patients survived during the period of observation. When selecting patients for transcriptome analysis, additional inclusion criteria were applied: age 55–80 years and male sex. As a result, 8 patients with severe COVID-19 were enrolled in the present study. The baseline demographic and clinical characteristics of the patients are summarized in Table 1.

The study was conducted in accordance with the World Medical Assembly Declaration of Helsinki: Ethical Principles for Medical Research Involving Human Subjects. All blood samples were collected at the time of admission with the informed consent of the investigated patients. The study was approved by the Ethics Committee of the Pavlov First State Medical University of St. Petersburg (Russia).

### 2.2. Isolation of Peripheral Blood Mononuclear Cells

Peripheral blood mononuclear cells (PBMCs) were isolated from 8 mL EDTA-anticoagulated venous blood by a Fi,coll-Paque gradient method (Ficoll-Paque PLUS, GE Healthcare, Chicago, IL, USA) [19].

After centrifugation, PBMCs were collected from the interface and washed twice with PBS (pH 7.4) to remove the platelet-rich plasma fraction. The PBMC cell pellets were aliquoted and resuspended in TRIzol Reagent (Thermo Fisher Scientific, Waltham, MA, USA) and immediately frozen at −80 °C.

### 2.3. RNA Isolation, Library Preparation, and Sequencing

Total RNA was extracted from PBMCs of SARS-COV-2 infected patients using TRIzol^TM^ Reagent (Thermo Fisher Scientific, USA) following the manufacturer’s protocol. All RNA samples were stored at −80 °C for subsequent analysis. RNA quality and quantity were analyzed using a NanoDrop spectrophotometer system (Thermo Fisher Scientific) and an Agilent 2100 Bioanalyzer (Agilent Technologies, Santa Clara, CA, USA) with the RNA 6000 Nano Kit according to the manufacturer’s instructions. All samples had an RNA integrity number (RIN) >8. Then, total RNA was used to obtain polyA fractions using oligoT magnetic beads Dynabeads mRNA Purification Kit (Ambion, Austin, TX, USA) according to the manufacturer’s protocol. Further, libraries for massive parallel sequencing were prepared from polyA RNA using a NEBNext Ultra II RNA Library Prep Kit (NEB, Ipswich, MA, USA) and NEBNext Multiplex Oligos for Illumina (Index Primers Set 1) according to the kit instructions. The concentration of the libraries was determined using Qubit dsDNA HS Assay Kit with a Qubit 2.0 Fluorometer (Thermo Fisher Scientific). The lengths of distribution fragments from the cDNA libraries were assessed on an Agilent 2100 Bioanalyzer (Agilent Technologies) using the Agilent High Sensitivity DNA Kit (Agilent Technologies). Unpaired (50 bp reads) sequencing of these libraries was performed on the Illumina HiSeq1500 platform using the TruSeq SBS Kit V3 sequencing reagents (Illumina, San Diego, CA, USA). RNA-Seq data for each sample (raw and processed data) can be accessed in the Gene Expression Omnibus, accession number GSE185863.

### 2.4. Data Processing and Analysis

**Figure 1:**
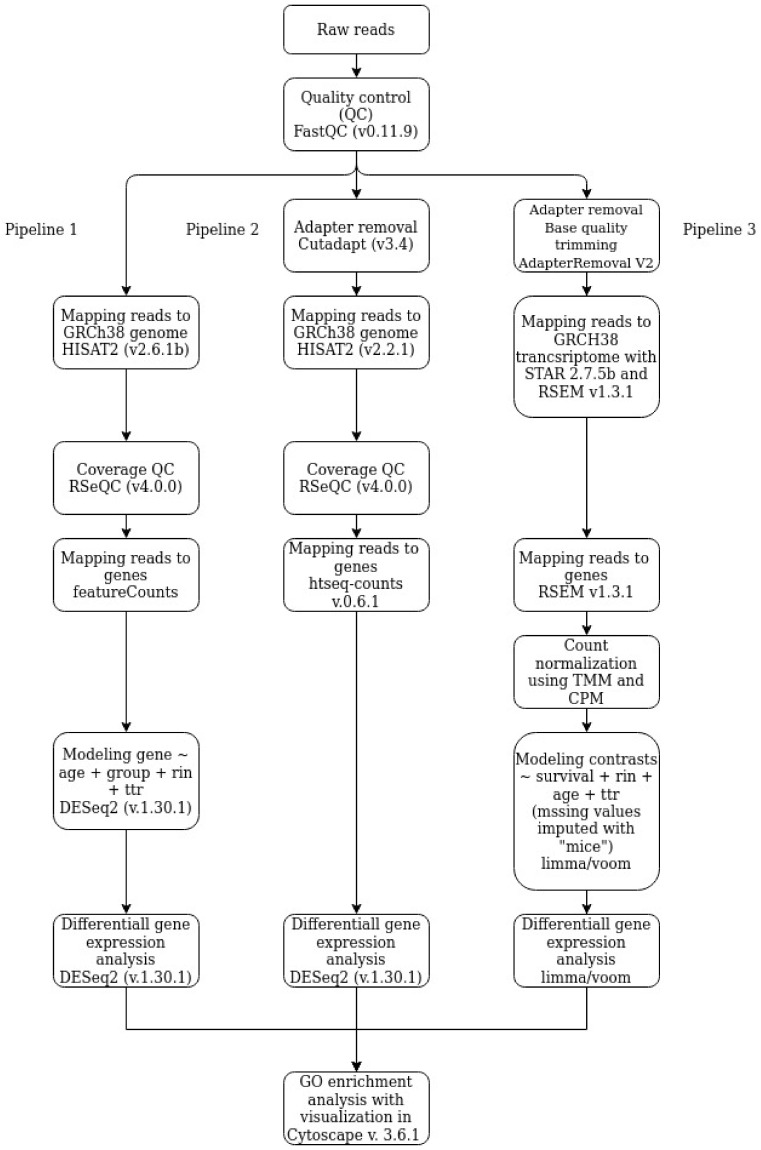
A comparative block diagram of pipelines for RNA-seq data processing and analysis.

RNA-seq data were analyzed to identify differentially expressed genes (DEGs) using three pipelines according to the block diagram in Figure 1. Gene ontology (GO) analysis of identified DEGs was performed to reveal enriched pathways.

#### 2.4.1. Pipeline 1 

Fastq files for each sample were aligned to the GRCh38 genome (Gencode, release 37) using HISAT2 (v2.6.1b) [20] with default parameters. Quality control for each sample was performed using FastQC (version 0.11.9) [21] and RSeQC (version 4.0.0) [22]. Counting reads was done using featureCounts [23]. Differential expression analysis was done using DESeq function from DESeq2 with default settings [24] in R (version 4.0.3). The following design was used in the analysis: gene ~ age + group + RIN + time tissue collection (ttr). Detected differential expression of genes was considered significant at a false discovery rate (FDR) <0.05 and a fold change (FC) threshold >1.5. The significance threshold was set to an adjusted *p* < 0.05.

#### 2.4.2. Pipeline 2

Human reference genome assembly GRCh38 (hg38) and gene model annotation files were downloaded directly from the Gencode website (https://www.gencodegenes.org/human/) (release 37, accessed on 1 December 2021). Adapters were removed by Cutadapt (version 3.4). HISAT2 (version 2.2.1) [20] was used with default parameters to build an index of the reference genome and mapping reads to the genome. Quality control for each sample was performed by FastQC (version 0.11.9) [21] and RSeQC (version 4.0.0) [21]. Counts of the number of sequencing reads mapping to each gene after the alignment step were conducted using the htseq-count function from the HTSeq framework (version 0.6.1) [25]. Gene differential expression analysis of two groups (two biological replicates per condition) was performed using the DESeq2 package (version 1.30.1) [24] in R (version 4.0.3). The following design was used in the analysis: gene ~ age + group + RIN + ttr. Detected differential expression of genes was considered statistically significant at an FDR < 0.05 and an FC threshold >1.5.

#### 2.4.3. Pipeline 3 

Ambiguous and low-quality bases were removed from the files obtained during the FastQ sequencing. The bases were removed with AdapterRemoval V2 [26]. The trimmed files were aligned to the human reference genome GRCh38 and GRCh38.92 gene annotation using RSEM with rsem-prepare-reference and rsem-calculate-expression commands [27] and the -star option was also used to generate STAR indices [28]. The resulting pseudo-counts were normalized using the TMM algorithm implemented in the R “edgeR” package, the calcNormFactors command [29] and the CPM algorithm implemented in the R “limma” package, with the “voom” command [30]. To identify differential expression, the normalized reads were processed using the commands “voom” (estimating the ratio of mean to variance, determining weights for observations), “lmFit” (creating a linear model describing observations), and “eBayes” (determining model parameters) from the R “limma” package [30]. In the linear model, the outcomes of the disease, age, time from the moment of tissue collection to isolation, and the RNA integrity index were considered. The missing values were imputed using the R “mice” package, with the “predictive mean matching” method [31]. DEGs were selected according to the following criteria: an FC threshold >1.5 and *p*-value moderated t test limma with FDR correction for multiple testing <0.05.

#### 2.4.4. Go Enrichment Analysis

Gene Ontology (GO) enrichment analysis with biological processes terms of [32] was performed using Clue GO version 2.5.5 [33] and Cluepedia version 1.5.5 [34] for Cytoscape version 3.6.1 and was conducted for DEGs obtained using three pipelines. Significantly enriched terms were selected using the one-sided hypergeometric test with FDR correction (*p* < 0.01). Term groups were selected by ClueGO based on the number of common genes/term (>40%). For enrichment, only terms of at least level 3 and no more than level 8 were considered, with which at least 3 DEGs were associated and with which the total number of DEGs associated was at least 4% of all genes associated with the term. All genes for which at least three reads were identified were used as a background for enrichment analysis.

## 3. Results

### 3.1. RNA-Seq Experiments

We obtained PBMCs from each patient with severe COVID-19: five patients who survived (survivors) and three patients who died from infection in the ICU (nonsurvivors). Variation across biological replicates was low with Spearman correlation values within like replicates for the two groups ranging from 0.86 to 0.89.

### 3.2. Differential Gene Expression Analysis Using DESeq2 and Limma/Voom

After applying a statistical filter (FDR < 0.05 and absolute FC > 1.5), we identified a total of 1038 DEGs (6.9% of the total expressed 15793; 448 upregulated and 550 downregulated genes) using Pipeline 1; 866 DEGs (5.1% of the total expressed 16,991; 399 upregulated and 467 downregulated genes) using Pipeline 2; and 516 DEGs (3.1% of the total expressed 16,278; 188 upregulated and 328 downregulated genes) using Pipeline 3 in survivors compared with nonsurvivors among patients with severe COVID-19 enrolled in this study. Lists of all DEGs of the three pipelines are available in Supplementary Table S1. It should be noted that when searching for DEGs, we took into account all reads related to a particular gene without isolating individual spliced mRNA variants.

### 3.3. Enrichment Gene Ontology (GO) Analysis

To identify biological pathways in COVID-19 systematically, we performed GO enrichment analysis of significantly up- and downregulated genes for DEGs identified in each pipeline. The functional modules were identified as the mutually overlapping gene sets clustered together and named using GO hierarchical structure terms. A GO term enrichment was conducted for these genes. We considered “metabolic process” terms with *p* < 0.05 (Bonferroni corrected hypergeometric test) and all types of GO-term-to-gene connections. Main modules in Pipeline 1 were identified, such as low-density lipoprotein (LDL) particle receptor activity (GO:0005041), leukocyte differentiation (GO:0002521), cargo receptor activity (GO:0038024); Pipeline 2: LDL particle receptor activity (GO:0005041), leukocyte differentiation (GO:0002521), cargo receptor activity (GO:0038024), lipoprotein particle receptor activity (GO:0030228), positive regulation of cell adhesion (GO:0045785), leukocyte activation (GO:0045321). In Pipeline 3, the identified processes were divided into two large clusters that were not connected with each other: cluster A (LDL particle receptor activity (GO:0005041), leukocyte differentiation (GO:0002521), cargo receptor activity (GO:0038024), dendritic cell apoptotic process (GO:0097048), myeloid leukocyte differentiation (GO:0002573), mononuclear cell differentiation (GO:1903131), regulation of dendritic cell apoptotic process (GO:2000668), dendritic cell differentiation (GO:0097028)), and cluster B (purinergic nucleotide receptor signaling pathway (GO:0035590).

**Figure 2:**
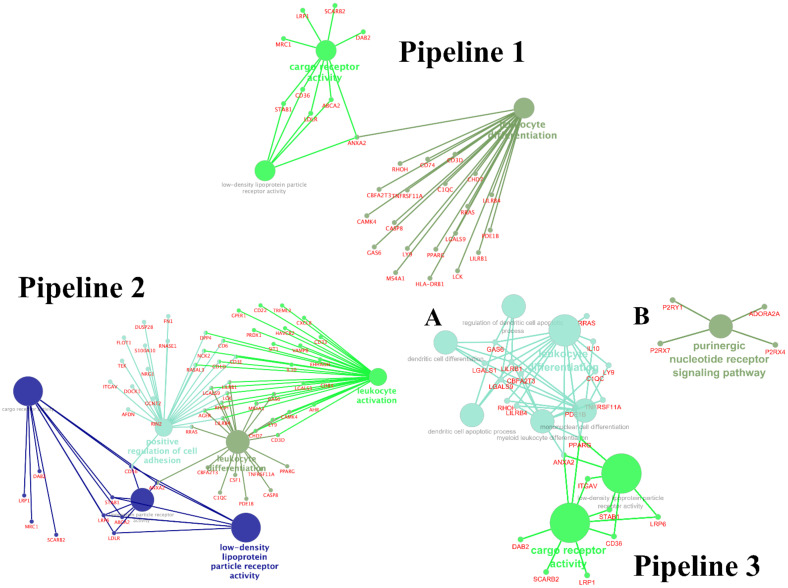
GO network analysis of the top enriched GO terms in the differently expressed genes between in survivors compared to nonsurvivors (multiple test correction by BH). Pipeline 1; Pipeline 2; Pipeline 3 with two clusters (Cluster A consists of the processes included in groups 1 and 2. Cluster B consists of the processes included in group 3).

Significant terms are presented in Figure 2 and Supplementary Table S2. Moreover, we found three overlapping enriched pathways between three pipelines, LDL particle receptor activity (GO:0005041), leukocyte differentiation (GO:0002521), cargo receptor activity (GO:0038024) between three pipelines in survivors compared with nonsurvivors (Figure 1, Supplementary Table S1). The DEGs involved in LDL particle receptor activity, cargo receptor activity, and leukocyte differentiation pathways in the three pipelines are presented in Supplementary Table S2. Interestingly, the determined gene sets included 62.5% genes from all pathway-associated genes of LDL particle receptor activity (GO:0005041) in Pipeline 1, 75% in Pipeline 2, and 46.16% in Pipeline 3 (Supplementary Table S2).

### 3.4. Enrichment Gene Ontology (GO) Analysis of Overlapping DEGs between Three Pipelines

**Figure 3:**
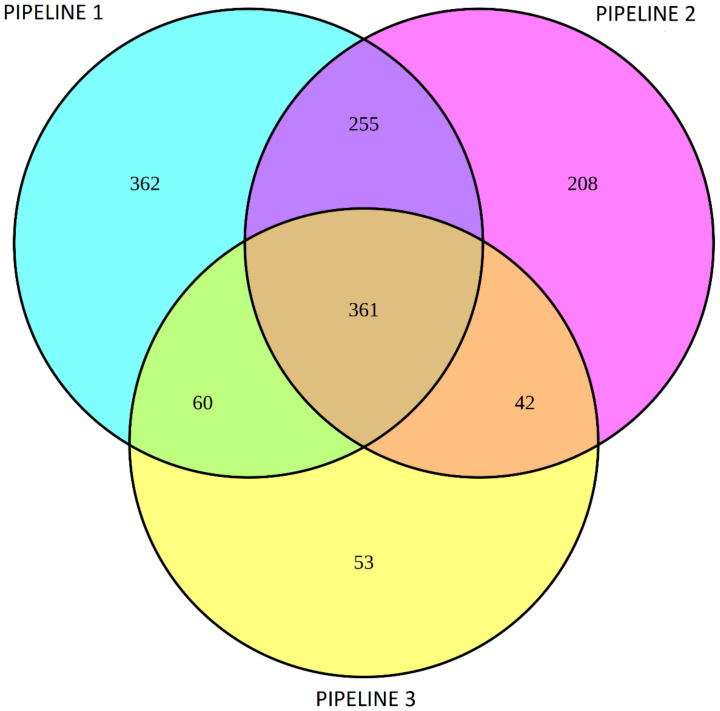
Venn diagram of differentially expressed genes in PBMCs of survivors compared to nonsurvivors among patients with severe COVID-19 between three pipelines.

Using a Venn diagram, 361 overlapping DEGs (248 upregulated, 113 downregulated) in PBMCs of survivors compared with nonsurvivors between the three pipelines were determined (Figure 3). The overlapping genes determined by the Venn diagram are presented in Supplementary Table S3. All the overlapping DEGs between the three pipelines had the same direction of fold change.

**Figure 4:**
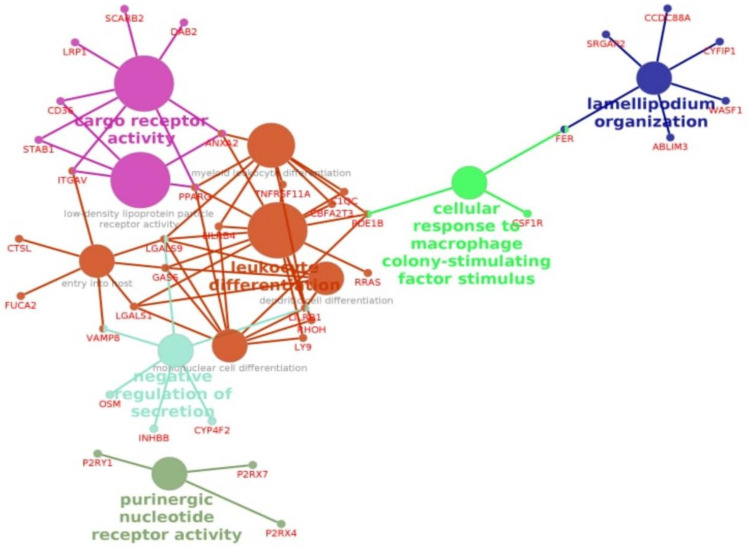
GO network analysis of the top enriched GO terms in the differently expressed genes between in survivors compared to nonsurvivors (multiple test correction by BH) in overlapping DEGs between all pipelines.

Next, we conducted GO analysis for 361 DEGs overlapping the three pipelines (Figure 4). The enrichment pathways, LDL particle receptor activity (GO:0005041), leukocyte differentiation (GO:0002521), and cargo receptor activity (GO:0038024), were also determined in this part of the analysis (Figure 4, Supplementary Table S4). For the selection of the putative target genes that may play a role in the outcome of COVID-19, we chose the genes that had at least two relations between three overlapping pathways (Table 2). We found downregulated expression levels of *STAB1*, *PPARG*, *CD36*, *ITGAV*, and *ANXA2* in survivors compared with nonsurvivors in all three pipelines.

## 4. Discussion

Here, we report for the first time to our knowledge, downregulation of LDL particle receptor pathway activity in patients of ICU surviving from severe COVID-19 infection. PBMCs mRNAs were assessed in patients with severe COVID-19 infection by whole transcriptome sequencing at the time of their admission to ICU. In this prospective study, we compared DEGs between different clinical outcomes (survival or nonsurvival) in the period of 30 days using the three pipelines. When DEGs were enriched into GO terms, several pathways were identified with the main two revealed in all bioinformatics models, namely the LDL particle receptor activity (GO: 0005041) and leukocyte differentiation (GO:0002521). The LDL particle receptor pathway activity was identified with a high degree of enrichment of up to 75% and was shown to be activated in nonsurvivors. This pathway is linked to the regulation of the cell uptake of cholesterol-rich LDL from the bloodstream [35].

During the past year, several studies have linked cholesterol metabolism with susceptibility to COVID-19 and the severity of the infection. First, a few studies showed that COVID-19 disease is accompanied by dyslipidemia [36,37]. Specifically, serum lipid levels decrease after acute COVID-19 onset and continue to decline in parallel with the increase of C-reactive protein concentration until the patient’s condition is resolved [37]. Moreover, serum LDL level is shown to be a predictor of poor disease prognosis [36,38,39]. In patients that did not survive, LDL levels decreased continuously until death and in one study were demonstrated to be reduced up to 60% compared with the level on admission [38]. Thus, one possibility is that downregulation of LDL particle receptor pathway activity in surviving patients might make them more resistant to consequences of decreased serum LDL levels.

Another important aspect of impaired cholesterol metabolism in COVID-19 may be associated with impaired lipid peroxidation processes and the accumulation of lipid peroxidation products, such as 4-hydroxynonenal, in deceased patients. This suggests that a poor prognosis in severe forms of COVD-19 may be associated with a decrease in antioxidant activity [40].

Another point is that during viral infection a critical role is played by specific cellular proteins determining virus–host interactions. Viral ability to enter the cell via ACE2, which is believed now to be a viral SARS-CoV-2 entry receptor, is supposed to be dependent on endogenic cholesterol synthesis. The hypothesis that the ACE2 receptor could shift out of the viral entry pathway under conditions of low cholesterol has been discussed [41,42]. Cholesterol-rich regions play an essential role in spike-mediated fusion, which is necessary for SARS-CoV-2 entry and replication [39]. Moreover, the involvement of cellular cholesterol homeostasis has been previously linked to viral entry and membrane fusion in the context of other infections, demonstrating a proviral function across different viral families [43,44]. It is interesting to note that a genome-wide CRISPR knockout screen using SARS-CoV-2 (USA/WA-1 isolate) in human cells revealed the involvement of genes linked to cholesterol metabolism as important for virus replication [45]. In addition, to support ACE2 as the distinct viral entry factor, resistant cell populations demonstrated downregulation of sterol regulatory element-binding protein (SREBP) and *NPC1*, the gene for Niemann–Pick intracellular cholesterol transporter 1, controlling the export of cholesterol from the endosomal compartment. These host factors could be critical for virus replication supporting infection because inhibiting cholesterol homeostasis pharmacologically could reduce the replication of SARS-CoV-2 [45]. It seems that SARS-CoV-2 needs endogenous cholesterol synthesis as an important host factor [45], and at the same time when entering the cell, it activates the family of SREBP transcription factors, which regulate lipid biosynthesis and sterol homeostasis. For example, a COVID-19 patient’s PBMCs expression of SREBP-2, which directly activates several genes involved in cholesterol metabolism, was highly activated [46]. Moreover, SREBF2 mRNA, sestrin 1 (SESN1), and proprotein convertase subtilisin/kexin type 9 (PCSK9) RNA levels were increased in PBMCs in COVID-19 patients in a severity-dependent manner.

It is interesting to note that in the present study, downregulation of *ANXA2* expression was also demonstrated in all pipelines in surviving patients. Annexin A2 (AnxA2) orchestrates multiple biological processes including vascular homeostasis, regulation of inflammation and immune system activation, tissue injury and repair, and cholesterol metabolism [47]. AnxA2 is reported to be an endogenous inhibitor of LDLR-degrading activity of PCSK9 and thus influences LDL-receptor and plasma cholesterol levels [48,49]. AnxA2 is also a recurrent host factor in a variety of viral infections [50]. It is unknown whether AnxA2 is implicated in SARS-CoV-2 infection or secondary viral infection, but its higher expression was associated with fatal outcomes in the present study.

All genes (*PPARG*, *CD36*, *STAB1*, *ITGAV*, and *ANXA2*) downregulated in surviving patients with COVID-19 are related to cholesterol homeostasis. *CD36* and *STAB1* both encode the scavenger receptors recognizing modified LDL particles [51,52,53]. CD36, first described as a platelet transmembrane glycoprotein, has now been shown to belong to a member of the scavenger receptor family class B [54] and is actively expressed in peripheral blood macrophages, where it plays an important role in innate immunity [55,56,57,58]. By contrast, this glycoprotein is also associated with the development of atherosclerotic lesions via its participation in the metabolism of oxidized LDL, phospholipids, and primarily fatty acids [59,60,61]. CD36 is a major scavenger receptor responsible for the recognition and internalization of oxLDL [51]. Receptor-mediated uptake of oxLDL by the monocyte-derived macrophages activates the reprogramming of innate immunity responses, termed “trained immunity” [51]. It is interesting to note that the representative network of blood transcriptional modules enriched in the monocytes of COVID-19 patients compared with controls, along with activation of T- and NK cells, cytopenia, and upregulation of cell cycle genes and immunoglobulins detected increased signals of monocyte activation including upregulation of *CD36* [16]. This may be because severe COVID-19 is characterized by a high frequency of classical monocyte subsets expressing CD36 [62]. We hypothesized that upregulation of CD36 expression could be a predictive factor for severe COVID-19 with possible lethal outcomes. Thus, a change in the CD36 gene expression can be associated with the unfavorable course of the disease in patients with a critical form of COVID-19 through at least two different mechanisms: activation of innate immunity to virus-containing cells and through activation of the atherogenesis processes via enhanced absorption of fatty acids and oxidized lipoproteins.

*STAB1* encodes a multifunctional scavenger receptor of an unusual type, which is actively expressed by tissue macrophages and sinusoidal endothelial cells; moreover, during inflammation, induction of stabilin expression was observed. STAB1 ensures exocytosis by macrophages of Gram-positive and Gram-negative bacteria, LDL (including oxidized and acetylated LDL), and advanced glycosylation end products [52,53]. We suppose that increased activity of *PPARG* as the key regulator of transcription activity of STAB1 and CD36 as receptors for modified LDL in the virus-infected areas may lead to greater lipid uptake and transient lipid depletion. This is consistent with the finding that serum lipid levels, namely total cholesterol, HDL-cholesterol, and LDL-cholesterol in patients with COVID-19 infection were significantly lower and associated with poor prognosis [63]. Several underlying mechanisms were suggested, including decreased LDL biosynthesis due to liver dysfunction, altered lipid metabolism because of acute inflammation, and/or lipid degradation induced by elevated free radicals [63].

In the present study, *PPARG* was upregulated in nonsurvivors. PPARγ can directly regulate lipid metabolism in immune cells (directly regulates the expression of genes involved in lipid transport and metabolism including *CD36*) [64]. Beyond regulation of classical lipid metabolism pathways, PPARγ activation may be involved in the presentation of lipid antigens to T-cells and modulation of the immune response through dendritic cells [64]. Thereby PPARγ bridges the lipid microenvironment and immune cell’s function driving its differentiation and functional phenotypes. PPARγ has an immunomodulatory function and plays a role in the resolution of inflammation, its upregulation in nonsurvivors may be a hallmark of nonresolved inflammation in condition of lipid depletion which is characteristic of severe COVID-19.

One common pathway for leukocyte differentiation was also identified, which indicates that the innate immunity and complement system, and T- and B-cell activation genes are dysregulated in nonsurvivors. Among genes identified in the three pipelines, those for growth arrest-specific protein 6 (GAS6), galectin-9 (LGALS9), and leukocyte immunoglobulin-like receptor B4 (LILRB4) were upregulated. Their plasma product levels are shown to be associated with disease severity. GAS6 plasma level is increased in COVID-19 patients compared with controls and decreased over time in patients surviving to 30 days post ICU admission [65]. Levels of galectin-9 and LILRB4 were increased from a control group to a mildly affected group, further increasing in severe and critical affected groups [66]. Interestingly, *LGALS9* is a PPARγ target and is upregulated via PPARγ overexpression [67].

Taken together, our findings suggest that induction of cholesterol metabolism and of LDL particle receptor pathway activity in patients with COVID-19 infection is associated with poor disease prognosis. It is worth noting that in accordance with our data in the recent publication of Meoni and coauthors, plasma metabolite and lipoprotein profiles in patients with COVID-19 demonstrated significant alterations compared with controls and was characterized with the lower levels of cholesterol and free-cholesterol HDL and LDL fractions. Importantly, treatment of toclizumab, a recombinant humanized monoclonal antibody against the interleukin-6 receptor, reverted methabolic alterations [68]. Taken together both RNA and metabolomics profiling conducted in the study cited above and our study support the close link between cholesterol homeostasis and susceptibility to COVID-19 and the disease severity. Inhibiting cholesterol biosynthesis may be a promising therapy for COVID-19.

The main limitation of the present study is the inclusion of only males and a small sample. Additionally, the serum lipids were not estimated in our patients in the course of the COVID-19 disease. Therefore, we did not know whether plasma lipid levels were different between patients in the survivor and nonsurvivor subgroups. To confirm the involvement of the cholesterol metabolism pathway in the progression of COVID-19, increasing the number of patients, and verifying the present findings with a simultaneous assessment of blood plasma cholesterol and lipoprotein levels appears warranted.

## 5. Conclusions

During the past year, several studies have linked cholesterol metabolism with susceptibility to COVID-19 and the severity of the infection and serum LDL level is shown to be a predictor of poor disease prognosis We report downregulation of LDL particle receptor pathway activity in patients of ICU surviving from severe COVID-19 infection and genes *PPARG*, *CD36*, *STAB1*, *ITGAV*, and *ANXA* was downregulated in surviving patients with COVID-19. All these genes are related to cholesterol homeostasis. For example, AnxA2 is reported to be an endogenous inhibitor of LDLR-degrading activity of PCSK9 and thus influences LDL-receptor and plasma cholesterol levels. *CD36* and *STAB1* both encode the scavenger receptors recognizing modified LDL particles. *STAB1* encodes a multifunctional scavenger receptor of an unusual type, which is actively expressed by tissue macrophages and sinusoidal endothelial cells; moreover, during inflammation, induction of stabilin expression was observed. We suppose that increased activity of *PPARG* as the key regulator of transcription activity of STAB1 and CD36 as receptors for modified LDL in the virus-infected areas may lead to greater lipid uptake and transient lipid depletion. This is consistent with the finding that serum lipid levels, namely total cholesterol, HDL-cholesterol, and LDL-cholesterol in patients with COVID-19 infection were significantly lower and associated with poor prognosis. In general, it seems that SARS-CoV-2 needs endogenous cholesterol synthesis as an important host factor, and our data confirm the important role of changes in cholesterol metabolism in determining the outcome of coronavirus infection.

## Figures and Tables

**Table 1:** Baseline demographic characteristics of the patients with COVID-19 at the time of admission.

ID Patients	Discharged Alive or Died	Age	Gender	T °C	Oxygen Saturation	Respiration Rate	CT-SKAN *	Therapy	Day of Death
38 34939	Dead	66	Male	37.1	65	26	4	Tocilizumab 400 mg CVVH 1470 min	28
98 36367	Dead	72	Male	37.5	88	22	2	Ruxolitinib 10 mg pd	19
101 37339	Dead	63	Male	36.9	80	25	4	Tocilizumab 400 mg CVVH 1520 min	30
114 37483	Alive	79	Male	36.7	92	22	3	Baricitinib 4 mg pd	-
51 35875	Alive	72	Male	38.0	92	22	4	Tocilizumab 400 mg CVVH 1440 min	-
96 36891	Alive	59	Male	37.6	86	27	4	Tocilizumab 400 mg	-
99 36444	Alive	63	Male	36.6	88	21	4	Tocilizumab 400 mg CVVH min 1490 min	-
126 37998	Alive	74	Male	36.8	89	22	4	Baricitinib 4 mg pd	-

* CT-SKAN scores depending on the extent of consolidation or ground-glass opacities: 1—<25%; 2—25–50%; 3—50–75%; 4—>75%.

**Table 2:** Differentially expressed genes in PBMCs from survivors compared to nonsurvivors involved in overlapping pathways with at least two relations between three overlapping pathways: low-density lipoprotein particle receptor activity (GO:0005041), leukocyte differentiation (GO:0002521), cargo receptor activity (GO:0038024).

Gene	Pipeline 1		Pipeline 2		Pipeline 3	
	log2FC	padj	log2FC	padj	log2FC	padj
*STAB1*	−1.67	5.75 × 10^−9^	−1.69	4.02 × 10^−9^	−1.83	5.15 × 10^−6^
*PPARG*	−1.68	0.001	−1.74	0.0045	−1.86	0.0007
*CD36*	−1.22	3.92 × 10^−5^	−1.25	5.47 × 10^−5^	−1.38	9.89 × 10^−5^
*ITGAV*	−0.93	0.0059	−1.17	0.0008	−1.15	0.0004
*ANXA2*	−1.15	4.83 × 10^−5^	−1.23	0.0004	−1.23	0.0002

## Data Availability

RNA-Seq data for each sample (raw and processed data) can be accessed in the Gene Expression Omnibus, accession number GSE185863.

## Supplementary Materials

The following are available online at https://www.mdpi.com/article/10.3390/cells10123495/s1. Supplementary Table S1, Lists of DEGs for all pipelines; Supplementary Table S2, Functional clusters for three pipelines; Supplementary Table S3, Overlapping DEGs determined by Venn diagram; Supplementary Table S4, Functional clusters for common DEGs.
