# Supplementary material for: Transcriptomic Profiles Reveal Downregulation of Low-Density Lipoprotein Particle Receptor Pathway Activity in Patients Surviving Severe COVID-19
Source: Cells. 2021 Dec 10;10(12):3495. doi: 10.3390/cells10123495 (PMC8700658; doi:10.3390/cells10123495)
Supplement: Supplementary file 1 [file cells-10-03495-s001.zip › cells-1457830-supplementary/cells-1457830-sp-1028/Supplementary Table 2_Functional clusters for three pipelines.pdf]

Supplementary Table S2. Functional clusters selected according to the results of the GO analysis

GO ID	GO term	padj	Associated Genes, %	Genes
**Pipepline 1**				
GO:0005041	low-density lipoprotein particle receptor activity	0,019	62.50	*ABCA2, ANXA2, CD36, LDLR, STAB1*
GO:0002521	leukocyte differentiation	0,015	14.79	*ANXA2, C1QC, CAMK4, CASP8, CBFA2T3, CD3D, CD74, CHD7, GAS6, HLA-DRB1, LCK, LGALS9, LILRB1, LILRB4, LY9, MS4A1, PDE1B, PPARG, RHOH, RRAS, TNFRSF11A*
GO:0038024	cargo receptor activity	0,0078	32.14	*ABCA2, ANXA2, CD36, DAB2, LDLR, LRP1, MRC1, SCARB2, STAB1*
**Pipepline 2**				
GO:0005041	low-density lipoprotein particle receptor activity	0,00030	75.00	*ABCA2, ANXA2, CD36, LDLR, LRP6, STAB1*
GO:0002521	leukocyte differentiation	0,0039	14.79	*AGER, ANXA2, C1QC, CAMK4, CASP8, CBFA2T3, CD3D, CHD7, CSF1, GAS6, LCK, LGALS9, LILRB1, LILRB4, LY9, MS4A1, PDE1B, PPARG, RHOH, RRAS, TNFRSF11A*
GO:0038024	cargo receptor activity	0,00036	35.71	*ABCA2, ANXA2, CD36, DAB2, LDLR, LRP1, LRP6, MRC1, SCARB2, STAB1*
(GO:0030228)	lipoprotein particle receptor activity	0,00439	54,55	*ABCA2, ANXA2, CD36, LDLR, LRP6, STAB1*
(GO:0045785)	positive regulation of cell adhesion	0,03722	11,71	*AFDN, AGER, CD1D, CD36, CD3E, CD6, DOCK1, DPP4, DUSP28, FLOT1, FN1, GCNT2, ITGAV, LCK, LGALS9, LILRB1, LILRB4, NCK2, NRG1, RASAL3, RHOH, RIN2, RNASE1, RRAS, S100A10, TEK*
(GO:0045321)	leukocyte activation	0,0281	10,53	*AGER, AHR, CAMK4, CD1D, CD22, CD33, CD3D, CD3E, CD6, CD84, CHD7, CXCL8, DPP4, EHHADH, GAS6,*

				GPER1, HAVCR2, IL10, LCK, LGALS3, LGALS9, LILRB1, LILRB4, LY9, MS4A1, NCK2, PRDX1, RASAL3, RHOH, SIT1, TREML2, VAMP8
**Pipepline 3**				
GO:0005041	low-density lipoprotein particle receptor activity	0,00015	46.15	*ANXA2, CD36, ITGAV, LRP6, PPARG, STAB1*
GO:0002521	leukocyte differentiation	0,0014	12.10	*ANXA2, C1QC, CBFA2T3, GAS6, IL10, LGALS1, LGALS9, LILRB1, LILRB4, LY9, PDE1B, PPARG, RHOH, RRAS, TNFRSF11A*
GO:0038024	cargo receptor activity	0,000022	31.03	*ANXA2, CD36, DAB2, ITGAV, LRP1, LRP6, PPARG, SCARB2, STAB1*
GO:0097048	dendritic cell apoptotic process	0,0107121	75,00	*GAS6, LGALS9, LILRB1*
GO:0002573	myeloid leukocyte differentiation	0,0335906	13,24	*ANXA2, C1QC, CBFA2T3, LGALS9, LILRB1, LILRB4, PDE1B, PPARG, TNFRSF11A*
GO:1903131	mononuclear cell differentiation	0,0238098	12,66	*GAS6, IL10, LGALS1, LGALS9, LILRB1, LILRB4, LY9, PDE1B, PPARG, RHOH*
GO:2000668	regulation of dendritic cell apoptotic process	0,0107121	75,00	*GAS6, LGALS9, LILRB1*
GO:0097028	dendritic cell differentiation	0,037209	33,33	*GAS6, LGALS1, LGALS9, LILRB1*
GO:0035590	purinergic nucleotide receptor signaling pathway	0,037209	33,33	*ADORA2A, P2RX4, P2RX7, P2RY1*
